# A transdiagnostic dimensional approach towards a neuropsychological assessment for addiction: an international Delphi consensus study

**DOI:** 10.1111/add.14424

**Published:** 2018-10-05

**Authors:** Murat Yücel, Erin Oldenhof, Serge H. Ahmed, David Belin, Joel Billieux, Henrietta Bowden‐Jones, Adrian Carter, Samuel R. Chamberlain, Luke Clark, Jason Connor, Mark Daglish, Geert Dom, Pinhas Dannon, Theodora Duka, Maria Jose Fernandez‐Serrano, Matt Field, Ingmar Franken, Rita Z. Goldstein, Raul Gonzalez, Anna E. Goudriaan, Jon E. Grant, Matthew J. Gullo, Robert Hester, David C. Hodgins, Bernard Le Foll, Rico S. C. Lee, Anne Lingford‐Hughes, Valentina Lorenzetti, Scott J. Moeller, Marcus R. Munafò, Brian Odlaug, Marc N. Potenza, Rebecca Segrave, Zsuzsika Sjoerds, Nadia Solowij, Wim van den Brink, Ruth J. van Holst, Valerie Voon, Reinout Wiers, Leonardo F. Fontenelle, Antonio Verdejo‐Garcia

**Affiliations:** ^1^ Brain and Mental Health Research Hub, Monash Institute of Cognitive and Clinical Neurosciences (MICCN) and School of Psychological Sciences Monash University Melbourne Australia; ^2^ Institut des Maladies Neurodégénératives Université de Bordeaux Bordeaux France; ^3^ Department of Psychology University of Cambridge Cambridge UK; ^4^ Addictive and Compulsive Behaviours Laboratory (ACB‐lab), Institute for Health and Behaviours University of Luxembourg Esch‐sur‐Alzette Luxembourg; ^5^ Department of Medicine Imperial College London UK; ^6^ Department of Psychiatry University of Cambridge; and Cambridge and Peterborough NHS Foundation Trust (CPFT) Cambridge UK; ^7^ Centre for Gambling Research at UBC, Department of Psychology University of British Columbia Vancouver BC Canada; ^8^ Discipline of Psychiatry, Faculty of Medicine, and Centre for Youth Substance Abuse Research The University of Queensland Brisbane Australia; ^9^ Alcohol and Drug Service, Royal Brisbane and Women's Hospital, Metro North HHS, Queensland Health and Discipline of Psychiatry The University of Queensland Australia; ^10^ Antwerp University (UA), Collaborative Antwerp Psychiatric Research Institute (CAPRI) Antwerp Belgium; ^11^ Department of Psychiatry the Sackler School of Medicine and Tel Aviv University Tel Aviv Israel; ^12^ Sussex Addiction Research and Intervention Centre, School of Psychology University of Sussex Brighton UK; ^13^ Departamento de Psicología Universidad de Jaén Spain; ^14^ Department of Psychology University of Sheffield Sheffield UK; ^15^ Institute of Psychology, Erasmus School of Social Sciences and Behavioral Sciences, Erasmus University Rotterdam the Netherlands; ^16^ Department of Psychiatry and Neuroscience Icahn School of Medicine at Mount Sinai NY USA; ^17^ Center for Children and Families, Department of Psychology Florida International University Miami FL; ^18^ Arkin Mental Health and Amsterdam UMC University of Amsterdam, Department of Psychiatry, Amsterdam Institute for Addiction Research Amsterdam Netherlands; ^19^ Department of Psychiatry and Behavioral Neuroscience University of Chicago Chicago IL USA; ^20^ Centre for Youth Substance Abuse Research The University of Queensland Brisbane Australia; ^21^ School of Psychological Sciences University of Melbourne Melbourne Australia; ^22^ Department of Psychology University of Calgary Calgary Canada; ^23^ Translational Addiction Research Laboratory Campbell Family Mental Health Research Institute, Centre for Addiction and Mental Health (CAMH) Toronto Canada; ^24^ Department of Family and Community Medicine, Pharmacology and Toxicology, Psychiatry University of Toronto Toronto Canada; ^25^ Neuropsychopharmacology Unit, Centre for Psychiatry, Division of Brain Sciences Imperial College London UK; ^26^ School of Psychology, Faculty of Health Sciences Australian Catholic University Melbourne Australia; ^27^ Department of Psychiatry Stony Brook University School of Medicine Stony Brook NY USA; ^28^ MRC Integrative Epidemiology Unit at the University of Bristol and UK Centre for Tobacco and Alcohol Studies, School of Experimental Psychology, University of Bristol Bristol UK; ^29^ Faculty of Health and Medical Sciences University of Copenhagen Copenhagen Denmark; ^30^ H. Lundbeck A/S Valby Denmark; ^31^ Departments of Psychiatry and Neuroscience, Child Study Center Yale University School of Medicine and Connecticut Mental Health Center and Connecticut Council on Problem Gambling New Haven CT USA; ^32^ Department of Neurology Max‐Planck Institute for Human Cognitive and Brain Sciences Leipzig Germany; ^33^ Cognitive Psychology Unit Institute of Psychology, and Leiden Institute for Brain and Cognition, Leiden University Leiden the Netherlands; ^34^ School of Psychology and Illawarra Health and Medical Research Institute University of Wollongong Wollongong NSW Australia; ^35^ The Australian Centre for Cannabinoid Clinical and Research Excellence (ACRE) New Lambton Heights NSW Australia; ^36^ Amsterdam UMC University of Amsterdam, Department of Psychiatry, Amsterdam Institute for Addiction Research Amsterdam Netherlands; ^37^ Department of Psychiatry University of Cambridge Cambridge UK; ^38^ Addiction, Development and Psychopathology (ADAPT)‐lab, Deptartment of Psychology University of Amsterdam the Netherlands

**Keywords:** Addiction, assessment, cognition, compulsions, decision‐making, habit, RDoC, reward, transdiagnostic

## Abstract

**Background:**

The US National Institutes of Mental Health Research Domain Criteria (RDoC) seek to stimulate research into biologically validated neuropsychological dimensions across mental illness symptoms and diagnoses. The RDoC framework comprises 39 functional constructs designed to be revised and refined, with the overall goal of improving diagnostic validity and treatments. This study aimed to reach a consensus among experts in the addiction field on the ‘primary’ RDoC constructs most relevant to substance and behavioural addictions.

**Methods:**

Forty‐four addiction experts were recruited from Australia, Asia, Europe and the Americas. The Delphi technique was used to determine a consensus as to the degree of importance of each construct in understanding the essential dimensions underpinning addictive behaviours. Expert opinions were canvassed online over three rounds (97% completion rate), with each consecutive round offering feedback for experts to review their opinions.

**Results:**

Seven constructs were endorsed by ≥ 80% of experts as ‘primary’ to the understanding of addictive behaviour: five from the Positive Valence System (reward valuation, expectancy, action selection, reward learning, habit); one from the Cognitive Control System (response selection/inhibition); and one expert‐initiated construct (compulsivity). These constructs were rated to be related differentially to stages of the addiction cycle, with some linked more closely to addiction onset and others more to chronicity. Experts agreed that these neuropsychological dimensions apply across a range of addictions.

**Conclusions:**

The study offers a novel and neuropsychologically informed theoretical framework, as well as a cogent step forward to test transdiagnostic concepts in addiction research, with direct implications for assessment, diagnosis, staging of disorder, and treatment.

## Introduction

The aetiopathogeny of addiction remains poorly understood, as we lack assessment models to identify vulnerability to addiction. Only 10–20% of patients with substance and behavioural addictions receive treatment 1, 2, 3, which tend to have modest outcomes, reflected in low compliance and high relapse rates 4. Thus, there is an urgent need for alternative assessment and intervention strategies to prevent or reduce the personal, social and economic burden associated with addictions.

Important developments in neuroscience have begun to reshape how addictions are understood 5, 6, 7. For instance, many individuals with addictions exhibit neuropsychological deficits across a range of functions subserved by reward, stress and cognitive‐control brain circuitries 8. These neuropsychological dysfunctions transcend traditional diagnostic boundaries and form a shared pathophysiological mechanism core to substance and behavioural addictions 9, 10, 11. Rapidly emerging evidence affirms that such mechanisms and processes result from dysfunction in frontal‐subcortical brain circuits 12, 13.

Key dysfunctions commonly shared across addictions include aberrant reward‐processing (e.g. inability to delay gratification; reward prediction error—the erroneous prediction of potential gains and losses associated with addictive behaviours) and increased stress sensitivity (e.g. elevated baseline stress levels; stress‐related cravings). These constructs may underlie reduced sensitivity to the negative consequences of addiction‐related actions (e.g. drug misuse or excessive betting) and have been associated with the development and later relapse of addictive behaviours 12, 14, 15, 16, 17, 18, 19, 20, 21. Other shared dysfunctions include impaired self‐control (e.g. reduced top–down, inhibitory control); linked to dysfunction in frontal‐subcortical brain circuits ascribed to decision‐making and goal‐directed behaviour 22, 23, 24, 25, 26, 27, 28, 29, 30, 31 which limit recovery 32, 33, 34, 35, 36. While there may not be a single phenotype, or set of related neural processes, that confers vulnerability to addictions, impairment in these reward, stress and control‐related processes may shape various pathways in and out of the addiction cycle.

Considering the above, superficially disparate (but conceptually related) disorders, such as substance‐use and gambling disorders, may be underpinned by overlapping neuropsychological processes and neural circuits. Such disorders may respond to similar interventions that target these common underlying mechanisms, such as naltrexone (an opioid‐receptor antagonist), which is effective in treating alcohol use disorder and gambling disorders putatively by targeting overlapping dysfunctional neurobiological systems 37, 38, 39. Synthesizing findings on effective treatments common to different substance and behavioural addictions would clarify shared mechanisms across addictive behaviours. It will also help to adjudicate whether a transdiagnostic approach is most appropriate, given alternative conceptualizations with the impulse control disorders and a putative compulsivity spectrum 40, 41. Importantly, the transdiagnostic approach highlights the clinical utility of targeting neuropsychological systems linked to disturbances in reward processing, stress reactivity and self‐control.

Nevertheless, with the exception of some developments such as cognitive‐bias modification 42, current approaches to clinical assessment and management have largely failed to integrate these developments into assessment and intervention tools. Two principal barriers to translation remain: (i) psychiatric assessment and diagnostic tools are based largely on characterization of symptoms (versus mechanisms), predicated on clinical reliability rather than biological validity, and based on self‐reports and observable behaviours rather than empirically measured dimensions; and (ii) neuropsychological assessments (as applied in the clinic) are based typically on paradigms developed decades ago for use in brain lesion cases and neurological disorders, which may lack sensitivity to the specific cognitive–emotional constructs key to the psychopathology of addiction.

To help address these shortcomings, the US National Institute of Mental Health (NIMH) developed the Research Domain Criteria (RDoC) initiative as a tool to encourage researchers to ‘…develop, for research purposes, new ways of classifying mental disorders’ beyond traditional nosologies, which were based on describing and counting overt signs and symptoms and arbitrary clinical thresholds and boundaries that encompass diverse and overlapping biological mechanisms 43. Biobehavioural dimensions captured by RDoC are measurable and linked to neural circuits and psychopathology; these are laid out as a series of matrices. Each matrix represents a functional domain that comprises several cognitive and affective processes, divided systematically into smaller subunits, each reflecting a specific measure of their corresponding construct. Contrary to the diagnostic classification system 44, the goal of this model is to use a data‐driven approach to determine constructs that aid in the understanding and classification of mental disorders. These classifiers are intended to serve as ‘intermediate phenotypes’, or neuroscientifically derived measures for improved biological modelling and targeted treatment interventions 45, 46.

The RDoC framework offers a neuroscientifically grounded approach to bridge clinical practice with neuroscience. It is operationalized via the RDoC matrix, which is designed to promote ongoing testing and refinement. 1Reflecting the evolving and dynamic nature of RDoC, changes were made recently made to the Positive Valence domain in late June 2018 (https://www.nimh.nih.gov/news/science‐news/2018/nimh‐releases‐updates‐to‐its‐rdoc‐framework.shtml). With regard to addiction, several constructs in the RDoC matrix could be used to conceptualize transdiagnostic processes implicated in such disorders. However, there is currently a lack of consensus on the discrete processes of the addiction cycle (i.e. initiation, regular use, impaired control, cessation, relapse) 47, 48, 49, 50, probably reflecting the different processes and phenotypes that interact at different stages of addictions and/or a lack of evidence‐based conclusions. For instance, the Positive Valence System is related to the early stages of addictive disorders, where drug use (for example) may lead to positive experiences (such as increased social bonding). Instead, the Negative Valence System might potentially be more relevant for avoidance of negative experiences (withdrawal symptoms) once drug‐seeking has become habitual and compulsive; the so‐called ‘end‐state’ of substance‐use disorders 51.

A common approach in mental health research to developing and refining research criteria, guidelines, reporting standards and protocols is the Delphi method 52. It is often used to capture practice‐based evidence, through an iterative process whereby a panel of topic experts is repeatedly surveyed until a consensus is reached among them (which may be ‘agreeing to agree’ or ‘agreeing to disagree’). In this study, we applied the Delphi method to synthesize expert opinion on which RDoC constructs and associated paradigms are most relevant to current understanding of addiction. The overarching aim of this large, international consensus study is to strengthen and integrate the knowledge gained from addiction neuroscience with clinical practice. A first step towards this goal is to develop a core assessment and classification protocol for substance and behavioural addictions through probing shared, key neuropsychological constructs, with the potential to improve health outcomes by allowing individuals to have their treatments tailored according to their underlying phenotype.

## Methods

### Expert panel

Recruited through purposive sampling, expert selection was based on being known to the research group (M.Y., A.C., L.F., A.V.G.), having relevant clinical and/or research experience or being internationally renowned experts in substance and/or behavioural addictions. A minimum of 5 years of professional experience and more than 50 scientific articles authored in peer‐reviewed journals were additional requirements. Using the procedure outlined by Okoli & Pawlowski 53, a work‐sheet was populated with potential experts who were subsequently categorized (i.e. field of expertise, profession, extent of clinical practice experience, number of publications, country and organizations), ranked and prioritized on the basis of both field of expertise and seniority in their area of expertise, and then sent invitations based on the target sample size. Although a sample of 20 has been deemed sufficient in the literature 54, 44 experts consented and 37 participated in the study. Expert views were surveyed online over three rounds (97% completion rate), with each successive round offering feedback for experts to revaluate their opinions. These experts were recruited from Australia (*n* = 8), Asia (*n* = 1), Europe (*n* = 18), North America (*n* = 9) and South America (*n* = 1). The study was approved by the local (Monash University) Human Research and Ethics Committee (CF15/3407–2 015 001 454).

### Procedure

**Figure 1:**
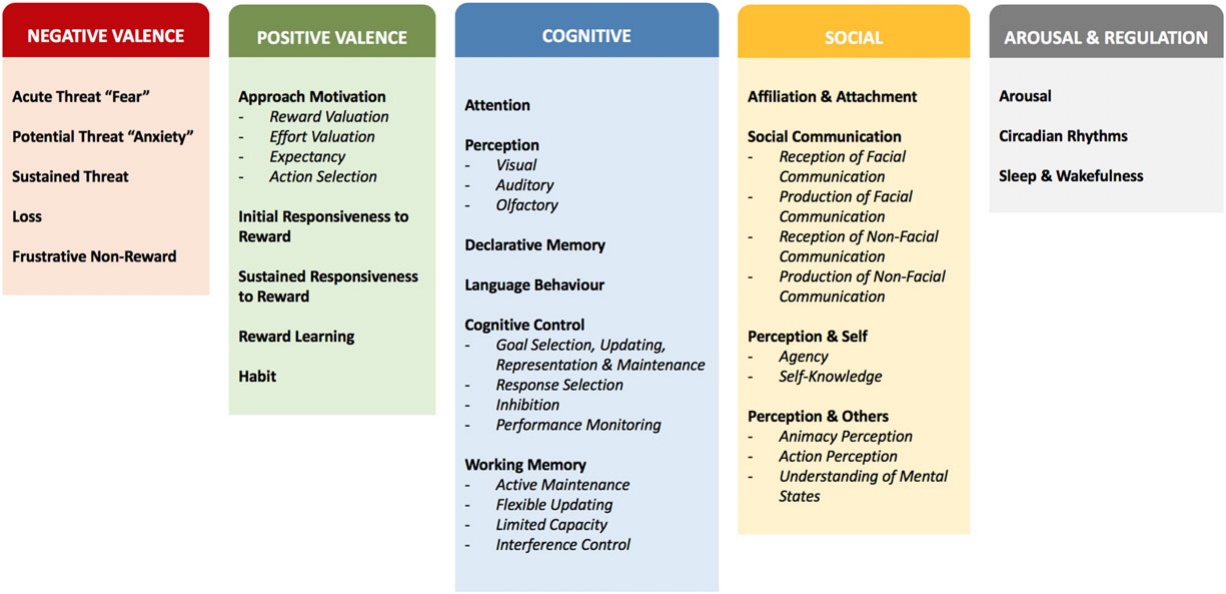
Overview of the Research Domain Criteria (RDoC) schema highlighting the five major domains, comprising 23 main constructs (bold text), wherein seven of these main constructs are further broken down into 23 subconstructs (italicized text), leading to a total of 39 primary and subconstructs. Note that in June 2018 (after the immediate completion of this paper), the Positive Valence domain of the RDoC matrix underwent a reorganization. The original constructs used in this study are mostly retained, but have been reorganized somewhat differently (see https://www.nimh.nih.gov/about/advisory‐boards‐and‐groups/namhc/reports/rdoc‐changes‐to‐the‐matrix‐cmat‐workgroup‐update‐proposed‐positive‐valence‐domain‐revisions.shtml)

Experts were required to participate in an online forum and rate the relevance of all 39 constructs of the RDoC to the concept of addiction (see Fig. 1). Although traditional Delphi studies commence with an open‐ended questionnaire, given the framework‐driven nature of our primary aim, the RDoC constructs formed the basis of the first‐round survey. To provide an opportunity for open‐ended responding and to generate a more complete item pool, experts were invited to suggest any additional constructs important in understanding addiction not delineated in the RDoC. After each round, constructs that did not achieve consensus moved into the subsequent round for re‐rating. These constructs were presented along with feedback outlining each expert's own previous response (blinded to other experts), the groups’ previous responses (percentages reflecting range and frequency) and a synopsis of all comments offered, regardless of whether the overall view was highly consistent or divergent. Provision of these comments afforded insight and rationale leading to a more accurate consensus, as opinion change is unlikely to occur without strong causal reasoning 55. To preserve an acceptable response rate of at least 70% across rounds 56, and to maintain rigour, identifiable data were disclosed to key researchers to follow‐up with non‐responders (up to three times each round). In the third and final round, experts who remained outside the consensus range were required to explain their rating in order to clarify their judgements 57.

## Data analysis

### Consensus and conclusion

A five‐point Likert scale ranging from 1 (unimportant) to 5 (essential) was used, with a non‐neutral midpoint of 3 (moderately important). Omitting a neutral mid‐point forces experts to deliberate and form an opinion, and where experts did not have the knowledge, a ‘don't know/unsure’ option was available as an addendum 58. Consensus was defined as ≥ 80% of experts endorsing a construct within two scale points 59, 60, 61. Constructs were excluded from the study if consensus fell between the lowest three scale points (‘unimportant’ to ‘moderately important’) and included as ‘primary constructs’ if consensus was achieved between the top two scale points (‘very important’ to ‘essential’).

The criterion for concluding the Delphi was not solely contingent upon reaching consensus, but also on the stability of responses 62, 63, allowing for any well‐defined disagreement to be maintained. The process was therefore deemed complete either when all items had achieved consensus or movement between rounds was less than 15%, indicating that opinions were not likely to change further 64.

### Quantitative analyses

SPSS (version 22) (IBM Corporation, Armonk, NY, USA, 2013) was used for all quantitative analyses. For the small percentage of missing data (2.7%), pairwise deletion was applied 65. Frequencies were calculated to assess consensus. Stability over rounds was assessed by the percentage of change 64 between rounds.

### Qualitative analyses

Experts’ comments underwent several stages of thematic analysis by the core committee (M.Y., A.C., L.F., A.V.G. and E.O., collectively), in order to process the data systematically, first by identifying categories and then by identifying common themes 66. Experts were asked to rate constructs in relation to key stages of addiction in general, namely ‘vulnerability’ (both proximal and distal predisposing factors leading to the development of addictive disorders) and ‘chronicity’ (the persisting and relapsing state of addiction). Specifically, comments were first coded as being either importance‐related and/or staging‐related and then, within these categories, comments were coded further based on their specificity; that is, rating (unimportant to essential), and/or staging (vulnerability, chronicity). The resulting matrix was then grouped into themes. Within these themes, comments were summated and reduced to eliminate repetition, with more informative, rational or well‐explained comments chosen, while retaining as much of the experts’ original wording as possible 67. As repetition of statements can increase same‐thinking and result in increased confidence in one's own opinion, all types of responses were included in order to challenge conventional thinking 55.

As suggested by Jorm 52, the additional constructs recommended by experts were evaluated by the research team (M.Y., A.C., L.F. and A.V.G.) to confirm they were: (i) not already covered by the survey (i.e. RDoC); (ii) within the scope of the study; and (iii) articulated clearly; where they were not, the research group reviewed and adjusted the description accordingly. These additional constructs were then added to subsequent rounds.

## Results

### Retention and characteristics of experts

Of the original 44 consenters, 37 experts completed round 1 of the Delphi questionnaires. Retention was very high, with 36 (97.3%) round 1 completers also completing both second‐ and third‐round surveys.

Experts who completed round 1 were aged 32–67 years [mean = 43.2, standard deviation (SD) = 8.95], with 67.5% (*n* = 25) being male. They represented a range of professions and academic disciplines (some multiple) including scientist/neuroscientists (54.1%; *n* = 20), psychiatrists (27.0%; *n* = 10), psychologists/clinical psychologists/neuropsychologists (34.3%; *n* = 13), other medical doctors (5.4%; *n* = 2) and pharmacologists (5.4%; *n* = 2). Their professional settings were primarily universities (81.1%; *n* = 30), hospitals (21.6%; *n* = 8) and out‐patient clinics (16.2%; *n* = 6), and the most commonly held academic titles were Professor (43.2%; *n* = 16), Associate Professor (21.6%; *n* = 8) and Research Fellow/Assistant Professor (24.3%; *n* = 9), with 89% (*n* = 33) of experts holding a PhD. Experts represented many areas of addiction (e.g. alcohol, cannabis opioids, gambling, internet), which supports our transdiagnostic approach.

### Expert consensus on functional domains

**Figure 2:**
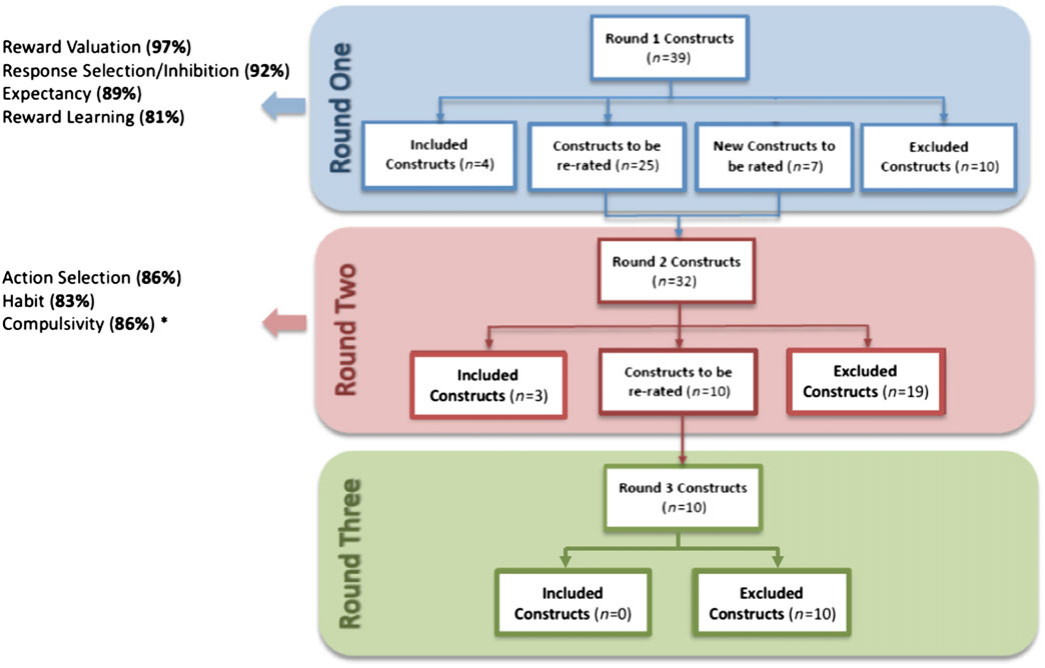
A flow‐chart of the constructs over each round highlighting items that were endorsed by ≥ 80% of experts as being clearly relevant (i.e. primary constructs; included items listed on the left together with percentage of experts endorsing the item), not relevant to addiction (excluded), created (i.e. new constructs, indicated by the asterisk), or re‐rated over the three survey rounds. [Colour figure can be viewed at wileyonlinelibrary.com]

**Figure 3:**
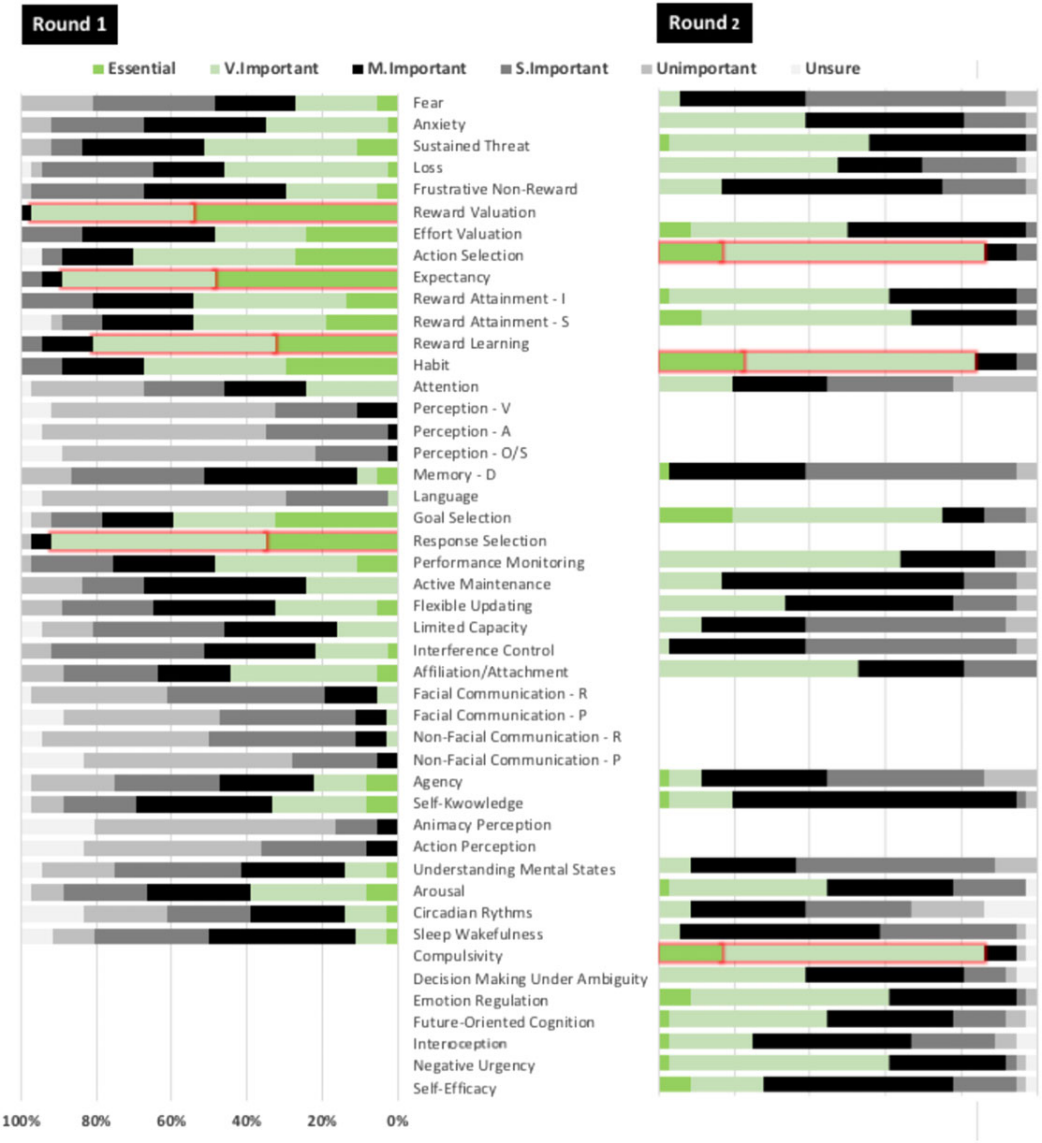
An overview of the consensus level and range for all 39 Research Domain Criteria (RDoC) (sub)constructs and seven additional constructs suggested by the experts for inclusion. All constructs were investigated over three rounds (only the first two rounds are shown, as the seven essential domains were derived in these rounds—all items in round three were excluded; percentages calculated relative to the total number reported). Note that expert‐suggested constructs were included in round 2 (bottom seven items in the list of constructs); the red highlight indicates the constructs that were selected as ‘Primary’ across the two rounds. V.Important = very important; M.Important = moderately important; S.Important = somewhat important; I = initial; S = sustained; V = visual; A = auditory; O/S = olfactory/somatosensory; D = declarative; R = reception; P = production; Expectancy = expectancy/reward prediction error; Action Selection = action selection/preference‐based decision‐making; Response Selection = response selection/inhibition

The consensus supported the inclusion of seven primary constructs, namely: (1) reward valuation; (2) expectancy/reward prediction error; (3) action selection/preference‐based decision‐making; (4) reward learning; (5) habit; (6) response selection/inhibition; and (7) compulsivity (see Fig. 2 for flow‐chart; Fig. 3 for an overview of the consensus level and range across the rounds for all constructs considered; and Table 1 for definitions). Table 1 summarizes the experts’ input on the neural circuits, physiological underpinnings and behavioural correlates of the primary constructs. Although this information was not analysed quantitatively, it provides a conceptual matrix consistent with the RDoC framework.

**Table 1:** Definitions of the seven ‘essential’ consensus domains, together with the relevant circuitry, self‐report and neuropsychological testing paradigms.

Construct	Definition	Circuits	Physiology/behaviour	Self‐reported examples	Cognitive paradigms	Expert commentary (selective)
Reward valuation	Processes by which the probability and benefits of a prospective outcome are computed and calibrated by reference to external information, social context (e.g. group input, counterfactual comparisons) and/or prior experience. This calibration is influenced by pre‐existing biases, learning, memory, stimulus characteristics and deprivation states. Reward valuation may involve the assignment of incentive salience to stimuli	Anterior medial OFC Corticolimbic circuits Ventral‐limbic striatum VTA/substantia nigra		BAS reward sensitivity subscale Sensitivity to reward subscale of the SRSPQ	Delay discounting probability choice task Willingness to pay task	‘…at the heart of addictive behaviours: if you are not sensitive to reward induced by the addictive behaviour, you won't develop that addiction’
Expectancy reward prediction error	A state triggered by exposure to internal or external stimuli, experiences or contexts that predict the possibility of reward. Reward expectation can alter the experience of an outcome and can influence the use of resources (e.g. cognitive resources)	Amygdala Basal ganglia Dorsal ACC Lateral habenula OFC Rostral medial tegmentum VTA/SN Ventral striatum	Cortical slow waves Heart rate change Skin conductance Goal tracking Pavlovian approach Reward‐related speeding Sign tracking	Affective Forecasting ASAM scale Eating expectancy inventory Generalized reward and punishment expectancy scale Self‐report of craving TEPS anticipatory scale	Drifting double bandit Rutledge passive lottery task Monetary incentive Delay task	‘Cue–reactivity and related constructs can play a role in escalation and maintenance of addictive behaviours. Reliable assessment is an issue, therefore not (yet) very suitable for diagnosis’
Action selection preference based decision‐making	Processes involving an evaluation of costs/benefits and occurring in the context of multiple potential choices being available for decision‐making	Amygdala			Balloon analogue risk task	‘Preference‐based decision‐making is probably most important for vulnerability (transition into problematic use). Diagnosis and chronicity are a bit more contested’
Reward learning	A process by which organisms acquire information about stimuli, actions and contexts that predict positive outcomes, and by which behaviour is modified when a novel reward occurs, or outcomes are better than expected. Reward learning is a type of reinforcement learning, and similar processes may be involved in learning related to negative reinforcement	Amygdala Dorsal striatum Medial pre‐frontal OFC Ventral striatum VTA/SN	Correct related negativity Error‐related negativity Feedback‐related negativity Midline theta Approach behaviours Consummatory behaviours Ecological momentary assessment	Ambulatory assessment and monitoring	Drifting double bandit Pavlovian conditioning Cambridge/Iowa Gambling Task Probabilistic reward task Probabilistic stimulus selection task Value‐modulated attentional capture task	‘Positive reinforcement is the key behavioural process behind initial drug (or other behaviour) exploration. Hence particularly relevant to initiation…’
Habit	Sequential, repetitive, motor or cognitive behaviours elicited by external or internal triggers that, once initiated, can go to completion without constant conscious oversight. Habits can be adaptive by virtue of freeing up cognitive resources. Habit formation is a frequent consequence of reward learning, but its expression can become resistant to changes in outcome value. Related behaviours could be pathological expression of a process that under normal circumstances subserves adaptive goals	Dorsal striatum Medial prefrontal SN/VTA Ventral striatum	Compulsive behaviours Repetitive behaviours Stereotypical behaviours	Aberrant behaviours checklist Measures of repetitive behaviours Self‐report habit index	Devaluation task Fruit task Habit learning task Habit task	‘“Unintentional” relapse related to shortened time‐period of “conscious” thought between stimulus/drug availability and use’
Response inhibition response selection	A subconstruct of the cognitive control system: that responsible for operation of cognitive and emotional systems, in the service of goal‐directed behaviour. This function is required when prepotent responses (those automatically elicited) are not adequate to meet the demands of the current context or need to be suppressed. Response inhibition has been presented in the literature as a facet of response selection, an executive process where one consciously withholds a response in the service of goal‐directed behaviour	DLPFC PPC VLPFC BA6/8(FEF) Pre‐SMA Ventral Frontostriatal	Alpha Gamma Theta Pupillometry Short interval cortical inhibition (TMS) Impulsive behaviours Distractibility Off‐task behaviours	BRIEF (Gioa) SANS/SAPS/PANSS ADHD rating scale (Dupaul) ATQ/CBQ effortful control Conners impulsivity scale Barratt questionnaire Impulsivity from UPPS	Flanker, Simon, Stroop Antisaccade Conflicting/contralateral motor response task Countermanding Go/NoGo Motor persistence paradigms Stimulus–response Incompatibility Stop‐signal reaction time	‘Inhibitory control is a foundational deficit in addiction, from substance use initiation to substance abuse treatment’ ‘….is a critical trait in risk of addictions and also shapes course of illness’
Compulsivity	This is the only additional construct to the RDoC received endorsement as a primary construct. In the present study, compulsivity was delineated as distinct from habit in that it can also be repetitive, or automatic behaviour. However, it is distinct from habit in that it can also be associated with negative outcome expectancy that contributes to the experience of being ‘forced’ or ‘compelled’ to act despite negative consequences, which further distinguishes it from impulsivity (the experience of being ‘driven’ and associated with positive outcome expectancies)	Dorsal striatum VLPFC DLPFC	Difficulties resisting urges and the experience of loss of voluntary control Repetitive behaviours performed in a habitual or stereotyped manner; inappropriate to the situation	Impulsive–Compulsive Behaviour Checklist CHI‐T YBOCS OCDUS Padua inventory OCI OCPD screener	Probabilistic reversal learning task Intra‐dimensional Extra‐dimensional set Shifting task Wisconsin card sorting task	‘Contributes to the subjective experience of “lack of control” that is part of the diagnostic criteria. The reported feeling of being unable to resist the desire to use undermines self‐efficacy and promotes relapse’

OFC = orbito‐frontal cortex; VTA = ventral tegmental area; VLPFC = ventrolateral prefrontal cortex; DLPFC = dorsolateral prefrontal cortex; BA = Brodmann's area; PPC = posterior parietal cortex; SMA = supplementary motor area; SN = substantia nigra; ACC = anterior cingulate cortex; TMS = transcranial magnetic stimulation; BAS = behavioural approach system; SPSRQ = sensitivity to punishment and sensitivity to reward questionnaire; ASAM = American Society of Addiction Medicine; TEPS = temporal experience of pleasure scale; BRIEF = behaviour rating inventory of executive function; SANS = scale for the assessment of negative symptoms; SAPS = scale for the assessment of positive symptoms; PANSS = positive and negative symptoms scale; ADHD = attention‐deficit/hyperactivity disorder; ATQ = adult temperament questionnaire; CBQ = children's behaviour questionnaire; UPPS = UPPS impulsive behaviour scale; CHI‐T = Cambridge–Chicago compulsivity trait; YBOCS = Yale–Brown obsessive–compulsive scale; OCDUS = obsessive compulsive drug use scale; OCI = obsessive–compulsive inventory; OCPD = obsessive compulsive personality disorder; RDoC = Research Domain Criteria.

### 1 Relevance of primary constructs to stage of disorder

**Figure 4:**
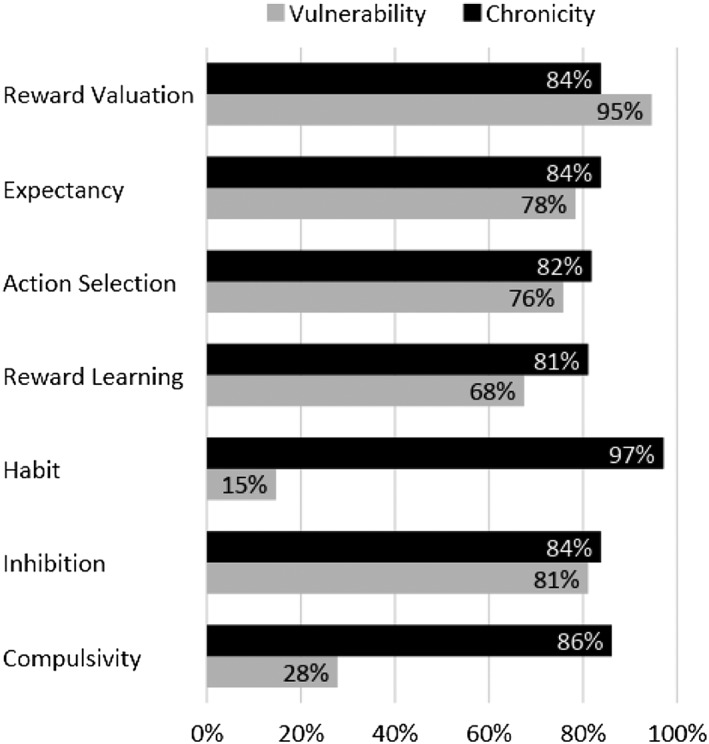
Experts’ endorsements for stages of disorder for primary constructs

As shown in Fig. 4, ‘reward valuation’ was considered the most relevant to vulnerability to addictions (consensus rating of 94.6%). In contrast, while all seven primary constructs were considered to be relevant drivers of chronicity, ‘habit’ and ‘compulsivity’ were seen to be selectively relevant to chronicity and least relevant to ‘vulnerability’ (habit: 14.7% vulnerability, 97.1% chronicity; compulsivity: 28.5% vulnerability, 86.1% chronicity).

## Discussion

Utilizing Delphi methodology, experts identified a circumscribed set of RDoC constructs, as well as other novel dimensions central to understanding substance and behavioural addictions. In total, seven constructs reached consensus as being primary constructs in understanding addiction, including RDoC reward valuation, expectancy/reward prediction error, action selection/preference‐based decision‐making, reward learning, habit and response selection/inhibition. Compulsivity is not described in the RDoC (at least as a monodimensional construct) but was introduced by experts. Considerable evidence exists supporting compulsivity as a core feature of addiction (although see 68), representing an ongoing and repeated difficulty in refraining from drug‐seeking or ‐taking despite negative consequences. It is worth noting that the Positive Valence domain of the RDoC matrix recently underwent a reorganization (published online 28 June 2018), where both habit and aspects of compulsivity (‘reward valuation’) have been expanded upon, which should help in their incorporation when studying addictions.

The high degree of consensus among experts across seven core constructs supports the proposition that there exists a group of common neuropsychological functions (and underlying neural processes) predisposing or maintaining addictive behaviours in individuals. These substrates primarily belong to the Positive Valence System in the RDoC matrix, which is noteworthy, as most neuropsychological assessment tools do not probe these functions thoroughly, focusing more upon cognitive skills (i.e. attention, memory, cognitive control and working memory). Much of the Positive Valence System research relies on neuroimaging methods and animal studies 69, highlighting the need for developing better, corresponding human behavioural measures. However, the findings align with empirically grounded neuropsychological models of addictive behaviours, including: (i) the incentive sensitization theory, emphasizing the link between aberrant reward learning and alterations in reward valuation 70; (ii) the Impaired Response Inhibition and Salience Attribution (I‐RISA) model, positing an imbalance between increased reward valuation/salience and deficient action selection/inhibitory control 13; (iii) the maladaptive habit‐learning model, proposing a transition between goal‐directed action selection and stimulus–response habits and compulsions 65; and (iv) decision‐making models, focusing upon how reward prediction and affective valuation influence preference‐based decisions 71, 72. There are robust practical and theoretical reasons to incorporate the five neuropsychological constructs from the Positive Valence System, plus the response inhibition and compulsivity constructs, in future research and clinical programs. Large‐scale addiction studies such as the US National Institutes of Health (NIH) ABCD study are already heading in this direction (see ABCDStudy.org).

From a research perspective, our findings stimulate the development of new addiction models seeking to integrate these constructs in a unifying framework that accounts for disorder staging. By contrast, a widely used model for describing addictive behaviours—the ‘dual systems’ approach, referring to an imbalance between reward valuation and the cognitive control systems 73—focuses upon only two of the Delphi‐identified constructs. However, some experts support a broader and more nuanced view 74, 75 in which many related, yet distinct, constructs (i.e. reward valuation, reward learning, preference‐based decisions, action selection, habits and corticostriatal neural systems) determine the expression of addictive behaviours. It is promising that the present consortium agrees with a high level of consensus on the essential constructs underpinning addictions.

Future research should delineate how these seven factors are independent or inter‐related. From a clinical perspective, a first step towards knowledge implementation is developing an assessment tool that measures these constructs validly and reliably. Along these lines, Kwako and colleagues proposed an assessment battery to target three primary domains (incentive salience, negative emotionality and executive functions) 76. The RDoC initiative is also contributing tasks towards research programmes whose goal is to collect data on dimensions relevant to mental health from a sample of 1 million or more individuals (https://allofus.nih.gov). Such large‐scale data collection efforts will help greatly in clarifying constructs broadly relevant to addictive behaviours and the mechanisms and processes relevant to various stages of addiction. Looking ahead, we need to develop an assessment battery that is time‐efficient, ecologically valid, psychometrically sound, sensitive to the seven primary domains identified herein, incorporates performance‐ and questionnaire‐based measures and is well tolerated.

### Relevance to staging of disorder

**Figure 5:**
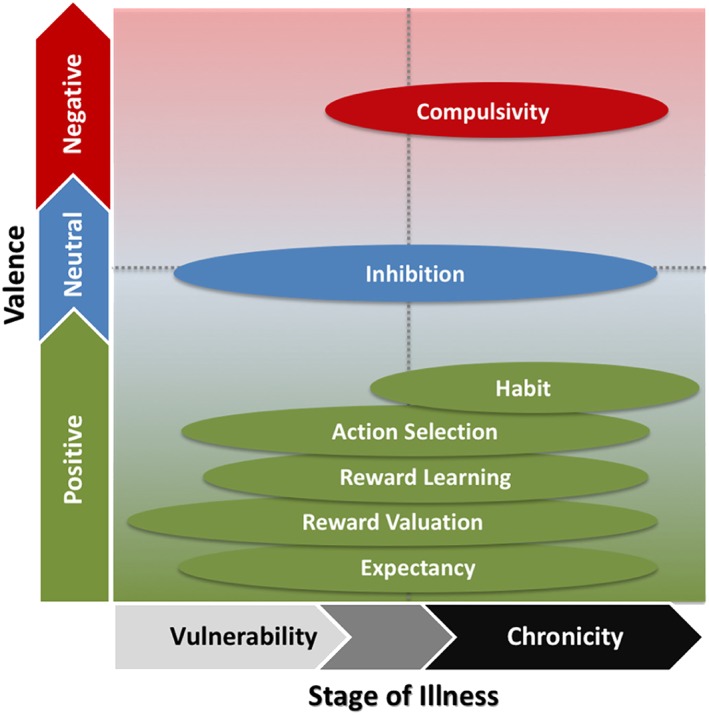
Expert‐endorsed primary constructs as a function of the major Research Domain Criteria (RDoC) domains (green = positive valence system; red = negative valance system; blue = cognitive system) and the constructs within these domains that are most relevant to the process of addiction (i.e. as a function of the relative size/width of the circles). Also illustrated are the relative influences of the seven primary constructs on the vulnerability to or the chronicity of addiction

Our findings raise the important issue of how the primary constructs (i) contribute to vulnerability to, or maintenance of, addictive behaviour; and (ii) predate addiction and emerge as a consequence of repeated drug use in vulnerable individuals. In relation to the former, aspects of the Positive Valence System, and the associated attribution of incentive salience to reward‐related stimuli, are considered important. For instance, at the vulnerability stage, reward valuation and linked anticipation may be a prominent factor in determining an individual's responsiveness to addiction‐related cues. At later stages of the addiction cycle, an allostatic‐incentive salience role of substance‐ or addiction‐related cues is likely to be present, and therefore reward valuation remains relevant to both vulnerability to relapse and chronicity. In relation to vulnerability to relapse (or chronicity), all seven primary constructs were considered relevant drivers (see Figs 4 and 5), but only ‘habit’ and ‘compulsivity’ were argued to be selectively relevant to chronicity.

Pre‐clinical data suggest that substance use may switch from being impulsive to compulsive over time, reflecting a shift from dopaminergic dysregulation of ventral to dorsal striatum function and related cortical, pallidal and thalamic circuitry 50, 77. Despite recent evidence that activation of the habit system during cue‐elicited tasks in humans is the best predictor of relapse 78, habit and compulsivity are two constructs highlighted in this Delphi study receiving the least human research to date. For instance, there has been little research investigating whether habits represent a gateway for the development of compulsivity 16, 29, and whether those with addictions show altered habit formation 79, impaired ability to disengage their habits in the face of negative consequences and, if so, whether habits can be updated and cognitive control retrained through intervention. Recent meta‐analyses confirmed habit‐related neuropsychological deficits in individuals with alcohol use disorder 80 and gambling disorder 81 compared to control participants. Beyond cue‐elicited tasks that relate partly to compulsivity (but not designed originally to encompass it), laboratory‐based models have been developed to assay habit learning, although these have yet to be applied widely to addictions (see 82 for a detailed review). Such experimental paradigms tend to be longer and more complex and may require approaches from growing fields such as computational psychiatry to optimize them for use in research and clinical settings. Validated clinical scales of compulsivity are also needed.

### Relevance to treatment and prevention and potential barriers to progress

Evidently, the relevance of many neuropsychological constructs are not constrained by traditional diagnostic boundaries, forming (at least partially) shared dysfunctions at the core of many substance and behavioural addictions. Established approaches to the clinical assessment and management of individuals with addictive disorders have not benefited fully from these emerging insights, with neuroscientists typically more aligned to the laboratory than the clinic. The essential neuropsychological dimensions currently identified provide a framework to guide clinicians and researchers through a consensual, collaborative agenda. Consistent with the RDoC framework, this agenda involves examining the diagnostic and prognostic value of dimensional measures of the constructs identified here, and the design of targeted, transdiagnostic treatment approaches to address these vulnerabilities and dysfunctions. The identification of neuropsychological targets may facilitate alternative interventions to succeed where others have failed. For instance, in the case of habits and compulsions (i.e. constructs related to chronicity), activities that re‐engage the cognitive control/goal‐directed systems (including mindfulness meditation or goal management strategies) may be effective in treating addictive behaviours 83. Regarding reward valuation, an individual who is vulnerable to placing a high value on addiction‐related hedonic experiences (e.g. substance use) may be at risk of developing an addiction. However, the same reward value system may also be protective if one can apply (or be treated to apply) their high reward value system to new forms of adaptive learning towards less harmful and more functional rewards or to distant rewards placing one towards a non‐use preference (see 84 for potential applications of this approach to contingency management, motivational interviewing/enhancement and targeted media campaigns). Such ‘redirecting’ approaches assume that the reward system is still fully operative and flexible, and thus malleable for ‘domain‐derived’ interventions 84. Accordingly, future research and clinical work can build upon the available neuroscience knowledge and be evidence‐based. The consensus‐derived knowledge from this paper thus provides a framework for grouping more homogeneous subtypes of addictions (currently classified in disparate categories), more validly linking disorder categories to molecular, cellular and neural dimensions, and guiding clinical interventions and treatments to core dimensions driving and maintaining addiction‐related disorders. A key additional advantage is the potential for prevention, as aberrant functioning in these systems can be detected well before first use of a substance and/or engagement in a maladaptive behaviour.

In relation to staging of illness, this neuropsychological approach underscores the frequent finding that many relevant phenomena vary continuously within and between addictions and mental disorders more broadly and in the population at large. These neuropsychological dimensions (may/arguably) become pathological at the extremes of an otherwise normal distribution 41. An online version of such an assessment battery could be used to measure and monitor potential risk factors for large cohort/population‐based studies with an eye towards early intervention.

### Limitations

Experts were only included if they were fluent English speakers. A handful of experts disagreed fundamentally with the use of the RDoC, arguing that they are too biological and reductionist, making the translation to phenomenological and other applications difficult 85. Indeed, many of the best currently available treatments are psychosocial in nature. Our findings need to be used to refine these approaches to create new psychosocial options that are more personalized and better targeted so as to improve current standards of assessment and care. Such views may have led experts to be less invested in the Delphi process, although the very high retention rate suggests otherwise. Other limitations relate to potential biases to our approach: (i) the research team promoting the Delphi have expressed publicly their views about addiction, which may have biased participants; (ii) although efforts were made to guarantee a broad representation of experts, the promoters may have introduced biases in the selection of experts; and (iii) finally, the pool of experts over‐represented European locations (versus the Americas and other non‐western countries) and academic positions (versus clinical practitioners), and thus results may be susceptible to biases related to prevailing views on addiction within Europe and academia.

## Conclusions

The theoretical framework established in the current study provides a platform to test predictions that: (1) the majority of individuals with substance and behavioural addictions have specific dysfunctions in the primary constructs identified by our International Expert Consortium; (2) these dysfunctions cut across diagnostic boundaries (i.e. individuals from different addictions will cluster into the same neuropsychological phenotypes); and (3) these indices can be linked differentially to vulnerability and chronicity (i.e. stage of disorder). This framework may enable grouping of more homogeneous disorder subtypes, better linking of behavioural questionnaire phenotypes to neural, cellular and genetic dimensions*,* guiding clinical decisions to the core issues that drive addictions and measuring the success and failure of treatment (i.e. providing a clinical end‐point). It is envisioned that the findings will guide and fast‐track the development of a new generation of neuropsychological assessment tools, and improve the monitoring and effectiveness of both established and future novel interventions.
